# PTFE Crystal Growth in Composites: A Phase-Field Model Simulation Study

**DOI:** 10.3390/ma15186286

**Published:** 2022-09-09

**Authors:** Ming Fan, Wenhao He, Qiangzhi Li, Jing Zhou, Jie Shen, Wen Chen, Yuanying Yu

**Affiliations:** State Key Laboratory of Advanced Technology for Materials Synthesis and Processing, School of Materials Science and Engineering, Wuhan University of Technology, Wuhan 430070, China

**Keywords:** phase-field model, polytetrafluoroethylene (PTFE)-based composites, polymer crystallization

## Abstract

We investigated, via a phase-field model simulation, the effects of a matrix’s properties and a filler’s characters on the polytetrafluoroethylene (PTFE) crystal growth process in composites under various supercooling degrees. The results show that the supercooling degree has a deciding influence on the crystal growth process. The intrinsic properties of PTFE polymer, such as anisotropic strength and phase transition latent heat, affect the growth rate, orientation, and interfacial integrity of the crystal trunk and the branching of the PTFE crystal growth process. The factors of the PTFE crystallization process, such as anisotropic strength and phase translation interface thickness, affect the uniformity and crystallization degree of the PTFE crystal. In the composites, the biphasic interface induces the crystal growth direction via the polymer chain segment migration rate, of which the degree depends on the shapes of the filler and the PTFE crystal nucleus. According to the results, choosing the low molecular weight PTFE and mixture filler with various particle sizes and surface curvatures as the raw materials of PTFE-based composites improves the crystallization of the PTFE matrix.

## 1. Introduction

Polytetrafluoroethylene (PTFE) is considered an outstanding candidate for use as a dielectric medium for high-frequency devices [1] due to its excellent dielectric properties and stability versus frequency, and it can meet the pressing demand for the rapidly developing modern information industry [2]. Although PTFE has excellent dielectric properties at a high frequency, its high thermal expansion coefficient, low thermal conductivity, and poor mechanical properties limit its application in electronic devices [3]. These deficits could be overcome by making PTFE-based composites with fillers to modify the matrix’s thermal, mechanical, and dielectric properties [2,4,5,6]. Researchers are trying to design composites by predicting the properties of the composites. For this purpose, it is the most popular way to analyze composite properties based on their composition. However, it does not work well most of the time since, besides the composition, the microstructure also determines the composites’ properties. After compounding with filler, the phase transition from the amorphous phase to the crystalline phase during processing progress is influenced by the biphasic interface greatly [7,8,9,10,11,12]. For composite materials, fillers have an impact on the crystallinity, microstructure characteristics, and local crystallization rate of polymer crystals [8]. The influence of compounds affects the nucleation and growth process of polymer crystals [9,10]. In addition to the common spherical and banded crystals of PTFE, fibrous crystals, shish-kebab crystals, and dendrites can be obtained with filler, extra field, or preparation processes [13,14,15,16,17,18,19]. With those, the crystal morphology and microstructure of the PTFE matrix can be tailored, and then, its properties, including thermal conductivity, dielectric loss, mechanical strength, and so on, are also affected [20,21,22,23,24]. This coupling effect between the microstructure and composite leads to a scale–span complexity [25,26,27], which brings difficulties and limitations to the experimental study of the composite properties. To overcome this barrier in composite material design and to clarify the action mechanisms of intrinsic and extrinsic factors on crystallization, it is necessary to clarify the influence of the biphasic interface on the PTFE crystallization process using computational simulation.

The phase-field method is an effective tool for investigating crystalline phase transformation. It builds phenomenological expressions describing physical systems based on the basic principles of thermodynamics and dynamics using an order parameter to distinguish the phases during solidification and avoiding the explicit tracking of the growth interface. It is a powerful way to predict microstructure evolution in the process of crystalline phase transformation, considering the microscopic, high-sharpness interface between two phases as the diffusion interface region in the computational domain, which conform to the non-equilibrium thermodynamic principle [28]. After Kyu’s pioneering work on the polymer crystallization phase-field model [29], the method was also developed and applicated in polymer research, covering the investigations of the crystal pattern formation, internal field generation during crystallization, and the nucleus’s effects [30,31,32,33,34,35,36]. However, as reported so far, there is no report on this method’s application in PTFE crystallization phase transformation.

In this paper, the Allen-Cahn phase-field equation [37] coupled heat transfer equation was used to simulate the crystallization phase transition process of PTFE. From the PTFE’s intrinsic properties to its crystallization process factors, we investigated the effects of the anisotropic mode, supercooling degree, anisotropic strength, latent heat, and interface thickness of PTFE crystal patterns. Furthermore, with the advantage of the phase-field method, we also studied the influence of different biphasic interfaces introduced by the second-phase filler and filler shape on the crystallization process of PTFE, to suggest a potential way to improve the performance of PTFE-based composites.

## 2. Methods

The crystallization process of PTFE was simulated using the phase-field method. In the model, the non-conserved order parameter, Ψ(***r***, t), is used to describe the phase evolution over time and space: Ψ(***r***, t) = 0 and Ψ(***r***, t) = 1. These denote the liquid phase and the complete crystalline phase, respectively. The total free energy of the system, *F*, consists of the local free energy density, *f*_local_ [38], and the gradient free energy density, *f*_grad_, which is Equation (1):

(1) $$F\left(\Psi ,T\right)=\int_{V}\left[f_{\mathrm{local}}\left(\Psi ,T\right)+f_{\mathrm{local}}\left(\Psi \right)\right]dV$$

A modified Allen-Cahn equation is used to describe the crystalline phase evolution, and its dimensionless form is shown in Equation (2):

(2) $$\begin{array}{cc} \frac{\partial \Psi (\hat{r},\hat{t})}{\partial \hat{t}}= & -\{W\Psi (\Psi -\xi )(\Psi -\xi_{0})-{\hat{\kappa }}_{0}^{2}\hat{\nabla }\cdot (\beta^{2}(\theta )\hat{\nabla }\Psi )\\ & +{\hat{\kappa }}_{0}^{2}\frac{\partial }{\partial \hat{x}}[\beta (\theta )\beta_{\Psi_{x}}^{\prime }(\theta ){\left(\hat{\nabla }\Psi \right)}^{2}]-{\hat{\kappa }}_{0}^{2}\frac{\partial }{\partial \hat{y}}[\beta (\theta )\beta_{\Psi_{y}}^{\prime }(\theta ){\left(\hat{\nabla }\Psi \right)}^{2}]\} \end{array}$$

where caret represents the dimensionless form, *W* describes the height of the nucleation barrier [32], *ξ* is a temperature-dependent unstable barrier [39], *ξ*_0_ is the value of Ψ in a stable solidification state [30,40], *κ*_0_ is the interface gradient coefficient (related parameters of the interface thickness). *β*(*θ*) = *1 + ε*cos(*jθ*) represents the interface anisotropic growth rate [41], where *ε* is the anisotropic strength, *j* is the number of modes, and *θ* is the angle between the interface normal and the reference axis.

The crystallization of the polymer is affected by the temperature field. At the same time, latent heat has an obvious influence on the growth of the crystal interface [30]. To determine the temperature at the growing crystal front, the heat conduction equation (which is coupled with the phase-field equation above) can be derived from the enthalpy conservation law, and its dimensionless form is shown in Equation (3):

(3) $$\frac{\partial \hat{T}}{\partial \hat{t}}=\hat{\alpha }{\hat{\nabla }}^{2}\hat{T}+\hat{K}\frac{\partial \Psi }{\partial \hat{t}}$$

where, *α = κ_T_*/(*ρC_P_*), Κ = Δ*H*/*C_P_* (related parameters of the latent heat); parameters *ρ*, *C_P_*, *κ_T_*, and Δ*H* represent density, specific heat capacity, thermal conductivity, and melting heat, respectively.

In this paper, Equations (2) and (3) are numerically solved on the grid region of the 600 × 600 square. The center finite difference method and explicit forward difference method are used for the discrete parts of space and time, respectively. The dimensionless time step and space step are fixed as Δ*t* and Δ*x* = Δ*y*, respectively. Cyclic boundary conditions are used for both phase-field variables and temperature fields at the boundary. Input parameters in the model are shown in Table 1. A detailed procedure for the derivation of the above equations, parameters, and dimensionless steps can be found in the Supplementary Information.

**Figure 1:**
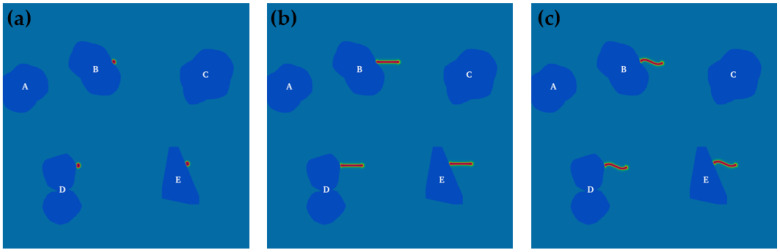
Shape design of the filler and the location of the crystal nuclei. (**a**) Circular crystal nuclei, (**b**) rodlike crystal nuclei, (**c**) curved crystal nuclei.

In addition, PTFE composite materials can be regarded as a large number of irregular fillers randomly distributed in a region. Using mathematics, we can find the appropriate description criteria, such as the pebble-shaped filler as a whole convex; the polygon filler’s angular surface has uneven characteristics in the mathematical description. Therefore, in this paper, the free deformation method, based on the stretch factor [45], is used to find the appropriate topological changes between the curves and obtain the parameterized representation of the filler. The shape of the curve can be controlled by changing the parameters interactively to better represent the actual filler shape. As shown in Figure 1, five kinds of fillers (A, B, C, D and E) are added to the simulated area. The shape settings of these fillers have three typical characteristics, namely, concave surface, convex surface, and flat plane (fillers A, B, C and D all contain convex and concave surfaces, and filler E corresponds to the flat plane). The attachment sites of the crystal nuclei are, respectively, arranged in these three places (concave position of filler B, convex position of filler D, flat position of filler E), and the rodlike and curved crystal nuclei are also arranged (as shown in Figure 1b,c).

## 3. Results and Discussion

### 3.1. Effects of Polymer Intrinsic Properties

In the phase-field model, the anisotropy function, *β*(*θ*), describes the anisotropy of crystal structures with various symmetries, including single crystal, spherulite, shish-kebab crystals, dendritic crystals, fibrous crystals, etc. In this paper, the crystallization transition processing of PTFE with three typical anisotropic modes (*j* = 4, 6, 36) [30,46], corresponding to square, hexagon, and circle, respectively, are simulated under various supercooling degrees, as shown in Figures S1–S3. The simulation results show that when *j* is 4 and 6, the overall shape of the PTFE crystal is a regular square and hexagonal, with high symmetry and a straight and clear boundary. Different from those two, when *j* = 36, the overall shape of the PTFE crystal is irregular and incomplete, and some crystals are separated from the main crystal under a low supercooling degree, as shown in Figure S3c; with the increase in the supercooling degree, the crystal shape is nearly round, and its boundary is relatively complete. When the supercooling degree is large enough, as shown in Figure S3k,o, a PTFE crystal with *j* = 36 shows orientation, and the branches of the longitudinal trunk grow preferentially toward the trunk. Under a low supercooling degree, the thermal movement of the polymer molecular chain is too intense to form a crystal nucleus, leading to low crystallization rate, which results in a small number of crystal branches, or even a large number of amorphous regions in the PTFE with *j* = 36. Comparably, a large supercooling degree promotes crystal nucleus formation and crystal growth, leading to the branches growing evenly and prolifically into a regular crystalline shape, which is consistent with the experimental preparation of dendritic PTFE crystals with a regular shape [16,17]. The branch and orientation of PTFE have a combinative influence on crystal morphology. The above results show that the orientation is determined by the intrinsic anisotropy of the model, while the growth of branches is affected by the supercooling degree.

**Figure 2:**
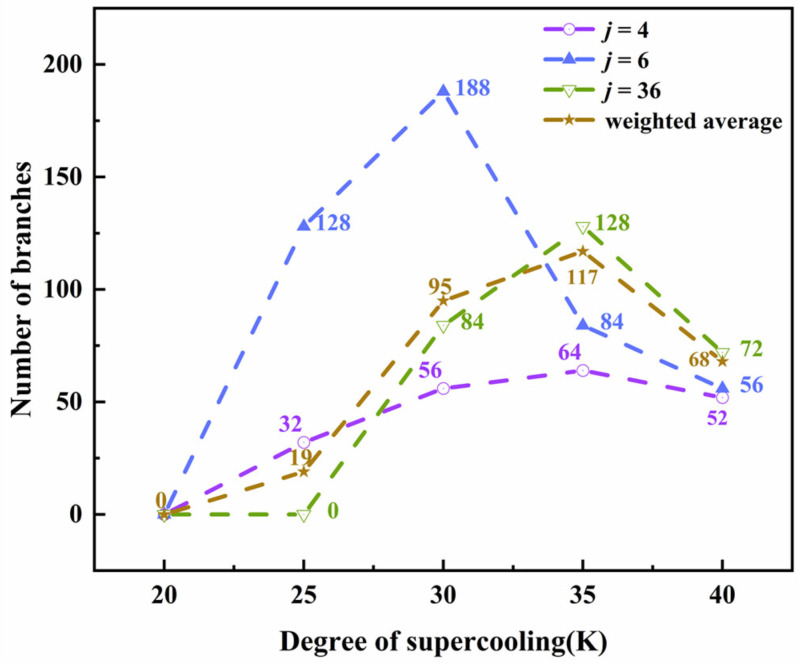
The relationship between the supercooling degree and the number of branches of a PTFE crystal with different anisotropic modes (evolution of time *τ* = 160).

To understand the mechanism clearly, Figure 2 shows the relationships between the number of branches and the supercooling degree. It can be seen that, in all three simulation systems with different anisotropic modes, the number of branches has the same varying trend. This reflects the competition between branch formation and crystal growth, both of which are driven by the supercooling degree. Under a low supercooling degree, the branches do not grow quickly, and there is enough space for branch formation. Thus, firstly, the number of branches increases with the increase in supercooling. However, with a further increase in the supercooling degree, the promoted growth rate makes the primary branches connect and fuse in a limited space, leading to the number of crystal branches decreasing and the branches becoming thick (as shown in Figures S1o, S2o and S3o). In three simulation systems with different anisotropic modes, the maximum number of branches in a PTFE with *j* = 6 appears under a lower supercooling degree. It is because there are more branch sites in this system than the one with *j* = 4, so a large number of branches can be differentiated quickly (the curve in Figure 2 shows a larger slope); however, the space between the trunk is small, and the number of branches is large, so the crystal branches fuse earlier, and the number of branches decreases quickly. Although the curves of *j* = 4 and 36 are similar, the phenomena are different. A large anisotropic mode, *j* = 36, leads to a small space in the trunk of the PTFE crystal, limiting the number of branches and resulting in an irregular crystal shape. In summary, the supercooling degree drives the crystallization process, while the anisotropy mode determines the branch sites and growth space of PTFE crystals. Both of these two factors determine the competition between the branch formation and crystal growth, whose results are the crystallinity and crystal shape of PTFE.

**Figure 3:**
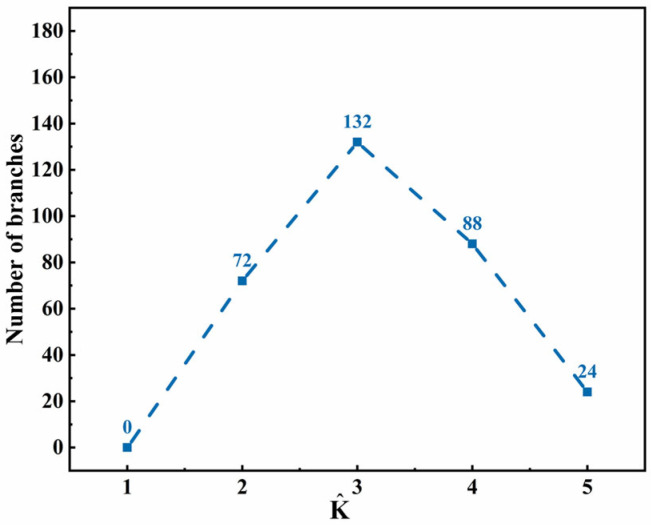
The relationship between the number of branches of a PTFE crystal and latent heat (anisotropic mode *j* = 6, evolution of time *τ* = 160).

In this paper, the heat conduction equation is coupled in the phase-field model, where the release of latent heat from the phase transition affects the entire free energy of the system, and, therefore, the influence of different dimensionless latent heats on the crystalline phase transition of PTFE has been simulated, as shown in Figure S4. Furthermore, the relationship between the number of branches and latent heat is shown in Figure 3. The simulation results in Figure S4 show that, when the latent heat is low, the trunk and branches of PTFE crystals become larger, and the closer the branches are to the trunk, the stronger their orientation along the trunk. With the increase in latent heat, the crystal growth slows and the crystal content (the area covered by the crystal in the simulated figure) decreases. As shown in Figure 3, with the increase in latent heat, the number of branches of the PTFE crystal increases first and then decreases. The mechanism of this variation is as follows. According to the dimensionless temperature distribution in Figure S4q,r, heat is preferentially released along the tips of each trunk and branch, which aggravates the thickening and orientation of a branch. However, the increase in latent heat slows this process down, and then PTFE crystals differentiate into more branches and secondary branches (Figure S4e–h), increasing the number of branches of crystals. With the further increase in latent heat, the total growth rate of the PTFE crystal slows down, and branches even grow in segments and discontinuities, as shown in Figure S4m–p. Then, the number of branches of the PTFE crystal decrease. Therefore, it is implied that a low latent heat release rate is conducive to a regular shape and uniform growth distribution of branches in terms of crystal morphology, and the path of latent heat release is correlated with the growth and orientation of branches.

### 3.2. Effects of Crystallization Process Factors

**Figure 4:**
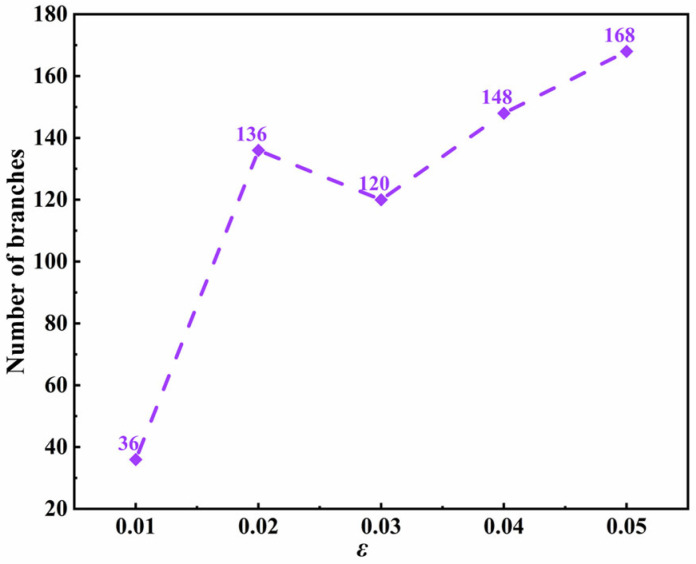
The relationship between the number of branches of a PTFE crystal and anisotropic strength (anisotropic mode *j* = 6, evolution of time *τ* = 160).

Anisotropic strength, as one of the important factors of crystallization processing, indicates the degree of surface tension and dynamic anisotropy at the solid-liquid interface of polymers. The anisotropic strength of the solid-liquid interface increases with the increase in liquid surface tension. The simulation results of the morphology of a PTFE crystal under various anisotropic strengths are shown in Figure S5. The results show that, with the increase in anisotropic strength, the size and shape of the trunk of the PTFE crystal remain almost the same, while the numbers and orientation degrees of the branches increase. This indicates that the crystal growth rate mainly depends on the supercooling degree, while the crystal morphology is sensitive to the anisotropic strength. Figure 4 describes the relationship between the number of branches and anisotropic strength. With the increase in anisotropic strength, the number of branches increases sharply then decreases slightly, and then it slowly increases. This is because the larger anisotropic strength amplifies the thermal disturbance at the interface, making the interface front unstable and eventually producing a more complex crystal morphology. Therefore, the compact and fine secondary branches of PTFE crystals are generated transversely, and their shapes are elongated along the longitudinal direction (Figure S5k,l,o,p). This also explains the slight decrease in the number of crystal branches: The number of secondary branches increases, but the primary branch on the longitudinal trunk decreases. It is indicated that the low molecular weight or the degree of polymerization of a PTFE, with low surface tension at the solid-liquid interface during the phase transition, will form a regular-shaped, uniform distribution of branches and a higher crystallization of the PTFE crystal.

**Figure 5:**
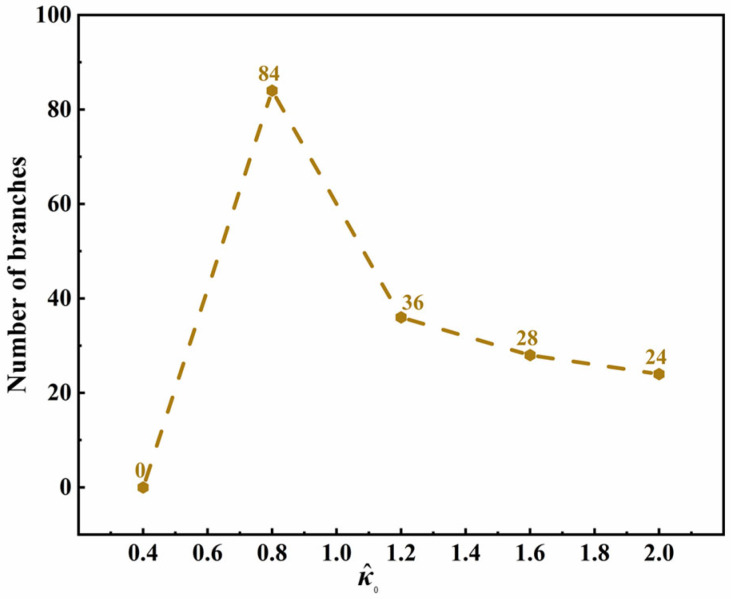
The relationship between the number of branches of a PTFE crystal and interface thickness (anisotropic mode *j* = 6, evolution of time *τ* = 160).

The advantage of the phase-field method for crystallization research is that it can deal with a dispersion phase-transition interface zone with a certain thickness, which makes the method close to ideal. In addition, it provides an effective tool to research the influence of interface thicknesses on the crystallization process. The crystal evolution of PTFEs with different interface thicknesses is simulated and shown in Figure S6. From the simulation results, the relationship between the interface thickness and the number of branches is found and shown in Figure 5. When the interfacial thickness is low, mass transfer between the solid phase and liquid phase is inhibited, and the crystal as a whole grows slowly, even without branching (Figure S6a–d). With the increase in interface thickness, mass transfer regions and paths increase, promoting PTFE crystal differentiation into uniform branches, and the degree of crystallization increases (Figure S6g). This effect leads to the number of branches increasing, as shown in Figure 5. However, when the interface thickness continues to increase, the isolation effect of the interface dispersion phase makes the branch growth discontinuous (Figure S6o), and the crystallization of PTFE decreases, as shown in Figure 5. Therefore, we suggest that the crystallization of PTFE can be improved by adjusting the interfacial thickness close to 80 nm by optimizing the molecular weight of the PTFE and the interfacial wettability, and high fluidity should be avoided.

### 3.3. Effects of Biphasic Interface and Crystal Nucleus Shape

**Figure 6:**
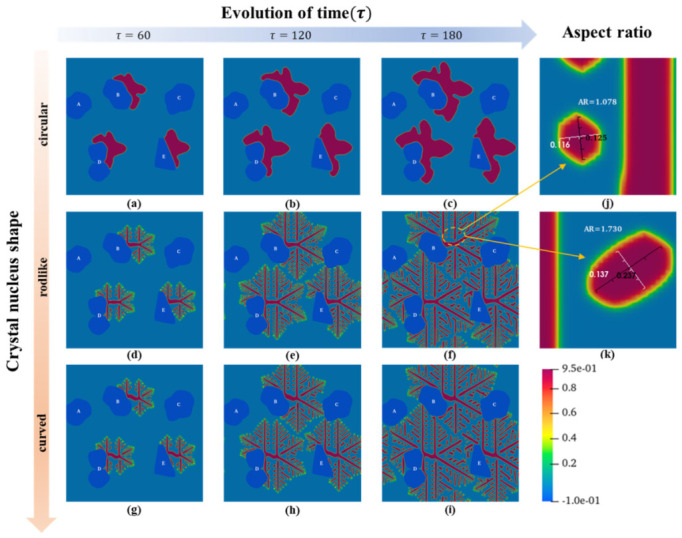
Morphology evolution diagram of a PTFE crystal with different nucleus shapes with a second-phase filler. (**a**–**c**) Circular crystal nucleus, (**d**–**f**) rodlike crystal nucleus, (**g**–**i**) curved crystal nucleus, (**j**,**k**) two examples of the defining aspect ratio (AR) for the effective branches.

Based on the study and analysis of the crystallization process of PTFE using the above phase-field model, the influence of the biphasic interface and crystal nucleus shape on the crystallization process of PTFE was studied. The simulation results of the growth of crystal nuclei with different shapes (circular, rodlike, and curved) at various biphasic interfaces (convex, concave, and flat) are shown in Figure 6. The growth mode of circular crystal nuclei (Figure 6a–c) is different from that of rodlike and curved crystal nuclei (Figure 6d–i).

**Figure 7:**
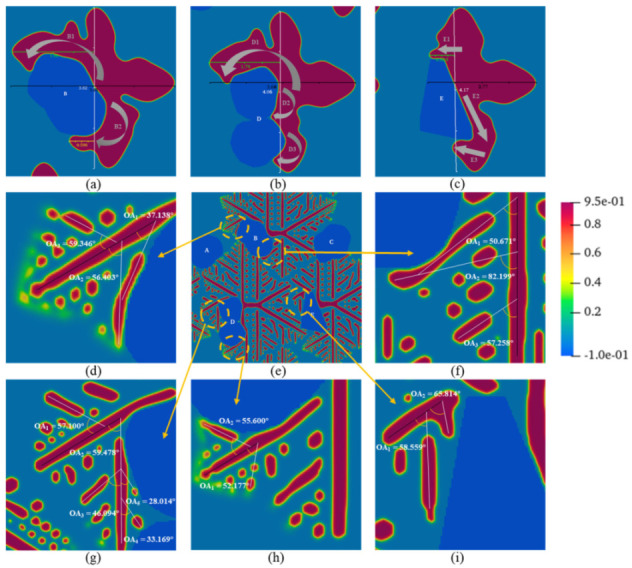
Details of PTFE crystal morphology at the biphasic interface of different fillers. (**a**–**c**) Details of Figure 6c; (**e**) morphology evolution diagram of a PTFE crystal with a rodlike nucleus shape with second-phase filler (evolution time *τ* = 180); (**d**,**f**–**i**) details of (**e**).

The crystal trunk of the circular crystal nuclei is thick but does not differentiate into branches. The shape of the crystal nucleus on the biphasic interface affects the overall shape of the PTFE crystal, and it can be seen that the crystal nucleus grows along the surface of the filler and gradually covers it. Figure 7a–c shows the enlarged details of the growth process of the circular crystal nuclei on the surfaces of different fillers in Figure 6c. The gray arrows reflect the local crystal growth trend. We found that the biphasic interface has no effect on the growth rate, but the variation in interface curvature, viz. the variation of the surface curvature of the fillers, leads to significant differences in the morphology of the PTFE crystal (comparing Figure 7a,b to Figure 7c). During the crystallization phase transformation of the polymer, the solid crystal continues to accept the polymer chain segment from the liquid phase for linear growth. This growth has no component change and only depends on the short-distance migration of the chain segment, while the short-distance migration rate of the chain segment near the interface is affected by the interface energy [47]. Therefore, the interface has a directional induction effect on the crystallization growth of PTFE, as the simulated results show.

The situations of rodlike and curved crystal nuclei are similar, as shown in Figure 6d–i. The nuclei differentiate into branches. Besides the initial orientation of the nuclei (as shown in Figure 1b,c), the trunks also grow along the interfaces, which is attituded to the directional induction effect of the interface. The results also show that the type of nucleus plays a decisive role in the morphology of PTFE crystals. 

To analyze the differentiation growth phenomenon in the composites, as shown in Figure 7d,f–i, the orientation angle (OA) between the trunks and effective branches of the composites with the rodlike nucleus is marked and measured in Figure 6f. The effectiveness of branch growth is judged by its aspect ratio (AR), and the critical value is AR ≥ 1.7, as shown in Figure 6j,k. From the variation in the different branches’ OAs, it is found that the branches near the interface are also bent by the directional induction effect of the interface. Because of space constraints, the concave and flat interfaces limit the growth of induced branches, as shown in Figure 7g,i. The convex interface promotes the growth rate of induced branches with the short-distance migration and provides an open space for their growth, indicating that ovoid and spherical fillers are preferred in achieving the high crystallinity of PTFE in composites.

## 4. Conclusions

The effects of the anisotropic mode, supercooling degree, latent heat, anisotropic strength, and interface thickness on the crystallization process and morphology of PTFE were studied by the phase-field model. The simulation results show that the anisotropic mode does not affect the overall growth rate of the crystal, but it does determine the shape and symmetry of the crystal. Increasing the supercooling degree and decreasing the molecular weight or degree of polymerization of PTFE is beneficial in obtaining regular and smooth PTFE crystals with more uniform distribution branches. In addition, the growth and local orientation of branches are related to the release rate and path of latent heat. On the basis of the above, the effects of biphasic interface and crystal nucleus shape on the crystallization of PTFE can be further studied. The simulation results show that the interface has a directional induction effect on the crystallization growth of PTFE. Furthermore, in the composites, the type of the nucleus determines the morphology of the PTFE crystal, and the shape of fillers influences the crystallinity of PTFE.

## Figures and Tables

**Table 1:** Sets of the material parameter and model parameter used for the simulations of PTFE [42,43,44].

Material Parameter	Model Parameter
$T_{m}=327\;℃$	$D=1\times 10^{-9}\;m^{2}/s$
$T_{m}^{0}=340\;℃$	$d=1\times 10^{-7}m$
$T_{c}=315\;℃$	$\xi_{0}=0.95$
$\rho =2.3\times 10^{3}\;{\mathrm{kg}/m}^{3}$	$\Delta \hat{t}=2.5\times 10^{-5}$
$\Delta H=8.2\times 10^{4}\;\mathrm{kJ}/\mathrm{mol}$	$\Delta \hat{x}=\Delta \hat{y}=1.5\times 10^{-2}$
$C_{p}=6.788\times 10^{4}\;\mathrm{kJ}/\left(\mathrm{mol}\cdot K\right)$	
$\kappa_{T}=0.256\;W/\left(m\cdot K\right)$	
$\sigma =1.86\times 10^{-2}\;{J/m}^{2}$	

## Data Availability

The data presented in this study are available upon request from the corresponding author.

## Supplementary Materials

The following are available online at https://www.mdpi.com/article/10.3390/ma15186286/s1, Figure S1: Morphology of PTFE crystal evolution under various supercooling degrees, *T_s_* (anisotropic mode *j* = 4). (a–d) *T_s_* = 20 K, *τ* = 40, 120, 200, 280; (e–h) *T_s_* = 25 K, *τ* = 40, 120, 200, 280; (i–l) *T_s_* = 30 K, *τ* = 40, 120, 200, 280; (m–p) *T_s_* = 35 K, *τ* = 40, 120, 200, 280, Figure S2: Morphology of PTFE crystal evolution under various supercooling degrees, *T_s_* (anisotropic mode *j* = 6). (a–d) *T_s_* = 20 K, *τ* = 40, 120, 200, 280; (e–h) *T_s_* = 25 K, *τ* = 40, 120, 200, 280; (i–l) *T_s_* = 30 K, *τ* = 40, 120, 200, 280; (m–p) *T_s_* = 35 K, *τ* = 40, 120, 200, 280, Figure S3: Morphology of PTFE crystal evolution under various supercooling degrees, *T_s_* (anisotropic mode *j* = 36). (a–d) *T_s_* = 25 K, *τ* = 40, 80, 120, 160; (e–h) *T_s_* = 30 K, *τ* = 40, 80, 120, 160; (i–l) *T_s_* = 35 K, *τ* = 40, 80, 120, 160; (m–p) *T_s_* = 40 K, *τ* = 40, 80, 120, 160, Figure S4: Morphology of PTFE crystal evolution with different dimensionless latent heat, $\hat{K}$. (a–d) $\hat{K}=2,\;\tau =40,\;80,\;120,\;160$; (e–h) $\hat{K}=3,\;\tau =40,\;80,\;120,\;160$; (i–l) $\hat{K}=4,\;\tau =40,\;80,\;120,\;160$; (m–p) $\hat{K}=5,\;\tau =40,\;80,\;120,\;160$; (q) dimensionless temperature distribution of Figure S4d; (r) local detail of Figure S4q, Figure S5: Morphology of PTFE crystal evolution with different aniso-tropic strengths, *ε*. (a–d) *ε* = 0.01, *τ* = 40, 120, 200, 280; (e–h) *ε* = 0.02, *τ* = 40, 120, 200, 280; (i–l) *ε* = 0.031, *τ* = 40, 120, 200, 280; (m–p) *ε* = 0.04, *τ* = 40, 120, 200, 280, Figure S6: Morphology of PTFE crystal evolution with different interface thickness related parameters, ${\hat{\kappa }}_{0}$. (a–d) ${\hat{\kappa }}_{0}=0.4,\;\tau =40,\;80,\;120,\;160$; (e–h) ${\hat{\kappa }}_{0}=0.8,\;\tau =40,\;80,\;120,\;160$; (i–l) ${\hat{\kappa }}_{0}=1.2,\;\tau =40,\;80,\;120,\;160$; (m–p) ${\hat{\kappa }}_{0}=1.6,\;\tau =40,\;80,\;120,\;160$ [30,31,38,39,40,41,48,49,50,51].

## References

[1] Xia Z.F., Wedel A., Danz R. (2003). Charge storage and its dynamics in porous polytetrafluoroethylene (PTFE) film electrets. IEEE Trans. Dielectr. Electr. Insul..

[2] Li Q., Liu P., Mahmood K. (2021). Improved properties of PTFE composites filled with glass fiber modified by sol-gel method. J. Mater. Sci. Mater. Electron..

[3] Apeldorn T., Keilholz C., Wolff-Fabris F. (2013). Dielectric properties of highly filled thermoplastics for printed circuit boards. J. Appl. Polym. Sci..

[4] Luan B.C.D., Huang J., Zhang J. (2015). Enhanced thermal conductivity and wear resistance of polytetrafluoroethylene composites through boron nitride and zinc oxide hybrid fillers. J. Appl. Polym. Sci..

[5] Peng G.R., Guo Z.L., Zeng P.Z. (2009). Study on glass fabric reinforced polytetrafluoroethylene composites infused with melted cyanate ester resin. J. Reinf. Plast. Compos..

[6] Atta A. (2021). Enhanced dielectric properties of flexible Cu/polymer nanocomposite films. Surf. Innov..

[7] Zhao W., Su Y., Müller A.J. (2017). Direct relationship between interfacial microstructure and confined crystallization in poly (ethylene oxide)/silica composites: The study of polymer molecular weight effects. J. Polym. Sci. Pt. B-Polym. Phys..

[8] Poerschke D.L., Braithwaite A., Park D. (2018). Crystallization behavior of polymer-derived Si-OC for ceramic matrix composite processing. Acta Mater..

[9] Li Y., Han C., Yu Y. (2018). Isothermal and nonisothermal cold crystallization kinetics of poly (l-lactide)/functionalized eggshell powder composites. J. Therm. Anal. Calorim..

[10] Liu Y., Qiu Z. (2020). Enhanced melt crystallization of biobased Poly (ethylene 2, 5-furandicarboxylate) by low loading of octavinyl-polyhedral oligomeric silsesquioxanes. Compos. Commun..

[11] Chang B., Schneider K., Lu B. (2018). Accelerating shear-induced crystallization and enhancing crystal orientation of isotactic-polypropylene via nucleating agent self-assembly. Polymer.

[12] Gohn A.M., Seo J., Ferris T. (2019). Quiescent and flow-induced crystallization in polyamide 12/cellulose nanocrystal composites. Thermochim. Acta.

[13] Wang J., Kim H.K., Shi F.G. (2000). Thickness dependence of morphology and mechanical properties of on-wafer low-k PTFE dielectric films. Thin Solid Films.

[14] Bosq N., Guigo N., Zhuravlev E. (2013). Nonisothermal crystallization of polytetrafluoroethylene in a wide range of cooling rates. J. Phys. Chem. B.

[15] Radusch H.J. (2005). Analysis of reversible melting in polytetrafluoroethylene. J. Therm. Anal. Calorim..

[16] Symons N.K.J. (1961). Crystals of polytetrafluoroethylene grown from solution. J. Polym. Sci..

[17] Melillo L., Wunderlich B. (1972). Extended-chain crystals. Colloid Polym. Sci..

[18] Khatipov S.A., Serov S.A., Sadovskaya N.V. (2012). Morphology of polytetrafluoroethylene before and after irradiation. Radiat. Phys. Chem..

[19] Laird E.D., Bose R.K., Wang W. (2013). Carbon nanotube-directed polytetrafluoroethylene crystal growth via initiated chemical vapor deposition. Macromol. Rapid Commun..

[20] Choy C.L. (1977). Thermal conductivity of polymers. Polymer.

[21] Choy C.L., Wong Y.W., Yang G.W. (1999). Elastic modulus and thermal conductivity of ultradrawn polyethylene. J. Polym. Sci. Pt. B-Polym. Phys..

[22] Dong S.Y., Zhu P., Liu J.G. (2019). Thermal Treatment Effects on the Microstructure and Tensile Properties of Transparent Polyamides. Acta Polym. Sin..

[23] Ding Y.C., Hou H.Q., Zhao Y. (2016). Electrospun polyimide nanofibers and their applications. Prog. Polym. Sci..

[24] Singh V., Bougher T.L., Weathers A. (2014). High thermal conductivity of chain-oriented amorphous polythiophene. Nat. Nanotechnol..

[25] Sung D.H., Kim M., Park Y.B. (2018). Prediction of thermal conductivities of carbon-containing fiber-reinforced and multiscale hybrid composites. Compos. Pt. B-Eng..

[26] Bian P.L., Verestek W., Yan S. (2020). A multiscale modeling on fracture and strength of graphene platelets reinforced epoxy. Eng. Fract. Mech..

[27] Fang W.J., Yang X.J., Li Q. (2020). Improved mode I interlaminar fracture toughness of random polypropylene composite laminate via multiscale reinforcing formed by introducing functional nanofibrillated cellulose. Compos. Pt. B-Eng..

[28] Moelans N., Blanpain B., Wollants P. (2008). An introduction to phase-field modeling of microstructure evolution. Calphad-Comput. Coupling Ph. Diagrams Thermochem..

[29] Kyu T., Chiu H.W., Guenthner A.J. (1999). Rhythmic growth of target and spiral spherulites of crystalline polymer blends. Phys. Rev. Lett..

[30] Xu H., Matkar R., Kyu T. (2005). Phase-field modeling on morphological landscape of isotactic polystyrene single crystals. Phys. Rev. E.

[31] Schultz J.M. (2012). Self-generated fields and polymer crystallization. Macromolecules.

[32] Wang X.D., Ou Y.J., Su J. (2013). A phase-field model for simulating various spherulite morphologies of semi-crystalline polymers. Chin. Phys. B.

[33] Wang X.D., Ou Y.J., Su J. (2014). Phase field modeling of the ring-banded spherulites of crystalline polymers: The role of thermal diffusion. Chin. Phys. B.

[34] Wang X.D., Ou Y.J., Su J. (2014). Investigating the role of oriented nucleus in polymer shish-kebab crystal growth via phase-field method. J. Chem. Phys..

[35] Wang X.D., Ou Y.J., Zhou W. (2016). A phase field technique for modeling and predicting flow induced crystallization morphology of semi-crystalline polymers. Polymers.

[36] Wang X.D., Ou Y.J., Liu Y. (2017). Prediction of Flow Effect on Crystal Growth of Semi-Crystalline Polymers Using a Multi-Scale Phase-Field Approach. Polymers.

[37] Liu F., Shen J. (2015). Stabilized semi-implicit spectral deferred correction methods for Allen-Cahn and Cahn-Hilliard equations. Math. Meth. Appl. Sci..

[38] Hohenberg P.C., Krekhov A.P. (2015). An introduction to the Ginzburg-Landau theory of phase transitions and nonequilibrium patterns. Phys. Rep. Rev. Sec. Phys. Lett..

[39] Harrowell P.R., Oxtoby D.W. (1987). On the interaction between order and a moving interface: Dynamical disordering and anisotropic growth rates. J. Chem. Phys..

[40] Costa A.R.D.M., Santos R.M., Ito E.N. (2019). Melt and cold crystallization in a poly (3-hydroxybutyrate)poly(butylene adipate-co-terephthalate) blend. J. Therm. Anal. Calorim..

[41] Wang X.D., Zhang H.X., Zhou W. (2017). A 3D phase-field model for simulating the crystal growth of semi-crystalline polymers. Int. J. Heat Mass Transf..

[42] Wu S. (2017). Polymer Interface and Adhesion.

[43] Mark J.E. (1999). Polymer Data Handbook.

[44] Xu H.J., Chiu H.W., Okabe Y. (2006). Breakup of spiral and concentric ringed spherulites in polymer crystallization. Phys. Rev. E.

[45] Wang X.P., Ye Z.L., Hu X.M. (2002). Shape modification and deformation of parametric surfaces. Comput. Aided Drafting, Des. Manuf..

[46] Karma A., Rappel W.J. (1998). Quantitative phase-field modeling of dendritic growth in two and three dimensions. Phys. Rev. E.

[47] Sun X., Xue B., Yang S.D. (2020). Structural conversion of PLLA/ZnO composites facilitated by interfacial crystallization to potential application in oil-water separation. Appl. Surf. Sci..

[48] Hohenberg P.C., Halperin B.I. (1977). Theory of dynamic critical phenomena. Rev. Mod. Phys..

[49] Landau L.D. (1936). The theory of phase transitions. Nature.

[50] Hoffman J.D., Weeks J.J. (1962). Melting process and the equilibrium melting temperature of polychlorotrifluoroethylene. J. Res. Natl. Bur. Stand. Sect. A.

[51] Housmans J.W., Peters G., Meijer H. (2009). Flow-induced crystallization of propylene/ethylene random copolymers. J. Res. Natl. Inst. Stand. Technol..

