# Supplementary material for: PTFE Crystal Growth in Composites: A Phase-Field Model Simulation Study
Source: Materials (Basel). 2022 Sep 9;15(18):6286. doi: 10.3390/ma15186286 (PMC9503715; doi:10.3390/ma15186286)
Supplement: Supplementary file 1 [file materials-15-06286-s001.zip › materials-1864456-supplementary.pdf]

materials

MDPI

Supplementary Information

PTFE Crystal Growth in Composites: A Phase-Field Model Simulation Study

1. Supplementary Methods

In the phase-field model, the non-conserved crystal order parameter, $\Psi(\boldsymbol{r}, t)$ (position $\boldsymbol{r}$, time t), is $\Psi = 0$ in the liquid phase and $\Psi = 1$ in the complete crystalline phase. According to Ginzburg–Landau theory [1,2], the evolution of $\Psi(\boldsymbol{r}, t)$, with time, is controlled by Equation (S1).

$$\frac{\partial \Psi(\boldsymbol{r},t)}{\partial t} = -\Gamma \frac{\delta F(\Psi,T)}{\delta \Psi(\boldsymbol{r},t)} \tag{S1}$$

where Γ is the interfacial mobility, T is the temperature, and $F(\Psi, T)$ is the total free energy functional of the system.

The total free energy of the system, $F(\Psi, T)$, consists of local free energy density, $f_{local.}$, and gradient free energy density, f_{grad}, as shown in Equation (S2).

$$F(\Psi,T) = \int_V \left[f_{\text{local}}(\Psi,T) + f_{\text{grad}}(\Psi) \right] dV \tag{S2}$$

where V is the region occupied by the system. According to the work of Kyu [3] and his colleagues, Ψ is defined as the ratio of the thin layer thickness, l, of the crystal to the optimal layer thickness, l_{opt}.

According to the Landau expression [4], the local free energy density, $f_{local}(\Psi, T)$, is, usually, an asymmetric double potential well function of Ψ and reaches two local minima in two stable states ($\Psi = 0$ and $\Psi = 1$). However, the crystal of the polymer is significantly different from other crystalline materials. Due to its complex long chain structure, the melt is difficult to crystallize completely and contains considerable defects or amorphous parts [5]. Therefore, when the polymer is in a stable solidification state, the order parameter, Ψ, should be less than 1. To describe the metastability of polymer crystals, Harrowel and Oxtoby [6] introduced the instability barrier, $\xi(T)$. The local free energy density, $f_{local}(\Psi, T)$, reaches a local maximum when $\Psi = \xi(T)$ between the local minima, $\Psi = 0$ and $\Psi = \xi_0 < 1$, where ξ_0 is the Ψ value in the stable solidification state depending on the degree of supercooling. Using these assumptions, a new local free energy density is shown in Equation (S3).

$$f_{\text{local}}(\Psi,T) = W\int_0^{\Psi} \varphi(\varphi - \xi_0)[\varphi - \xi(T)]d\varphi \tag{S3}$$

where W is the dimensionless coefficient describing the height of the barrier on the surface. The ξ_0 value can be estimated as $\xi_0 = T_m / T_{0_m}$ [7], since the degree of defect in polymer crystallization usually depends on the supercooling temperature, with T_m and T_{0_m} being the experimental melting temperature and the equilibrium melting temperature, respectively. In practice, the crystallinity, crystal morphology, and crystal defects of polymer solidification are closely related to supercooling. Thus, T_m may depend on supercooling. However, for each crystallization temperature, its value is often not determined experimentally. In this work, T_m is estimated with T_c and T_{0_m} according to the Hoffman–Week theory [8,9]. Due to the local free energy density, $f_{local}(\xi_0, T) < 0$ and $f_{local}(\xi(T), T) > 0$, there exists an $\hat{\Psi}$ such that $f_{local}(\hat{\Psi}, T)$ is 0, and Equation (S4) can be obtained:

$$\xi = \frac{4\xi_0\hat{\Psi} - 3\hat{\Psi}^2}{6\xi_0 - 4\hat{\Psi}} \tag{S4}$$

According to the Hoffman–Week theory, $\hat{\Psi}$ has the form shown in Equation (S5).

$$\hat{\Psi} = \frac{T_m^0 - T_m}{T_m^0 - T}\xi_0 \tag{S5}$$

In addition, the gradient of the crystal order parameter constitutes the gradient free energy density, $f_{\text{grad}}(\Psi)$, which is usually used to describe the symmetric or asymmetric growth process of crystal interfaces. Its form is as shown in Equation (S6):

$$f_{\text{grad}}(\Psi) = \frac{1}{2}\kappa_0^2\beta^2(\theta)(\nabla\Psi)^2 \tag{S6}$$

where κ_0 is the interface gradient coefficient and $\beta(\theta)$ is the interface anisotropic growth rate. In a 2D simulation, $\beta(\theta) = 1 + \varepsilon\cos(j\theta)$, where ε is the anisotropic strength, j is the number of modes, and θ is the angle between the interface normal and the reference axis.

Substituting Equations (S2) to (S6) into Equation (S1), Equation (S7) is obtained as follows:

$$\begin{aligned}\frac{\partial\Psi(\boldsymbol{r},t)}{\partial t} &= -\Gamma[W\Psi(\Psi-\xi)(\Psi-\xi_0) - \kappa_0^2\nabla\cdot(\beta^2(\theta)\nabla\Psi) \\ &+\kappa_0^2\frac{\partial}{\partial x}(\beta(\theta)\beta'_{\Psi_x}(\theta)(\nabla\Psi)^2) - \kappa_0^2\frac{\partial}{\partial y}(\beta(\theta)\beta'_{\Psi_y}(\theta)(\nabla\Psi)^2)]\end{aligned} \tag{S7}$$

Model parameters W and κ_0 are calculated from experimentally measurable quantities [10] in Equations (S8) and (S9).

$$W = 6\frac{\Delta H}{RT\xi_0^3}(\frac{T_m - T}{T_m^0})(\frac{\xi_0}{2} - \xi)^{-1} \tag{S8}$$

$$\kappa_0 = 6\frac{\sigma}{nRT}(\frac{2}{W})^{1/2} \tag{S9}$$

where ΔH is the melting heat, R is the gas constant, σ is the surface free energy per unit area, and n is the amount of substance per unit volume of the polymer monomer.

The crystallization of the polymer is affected by a temperature field produced by latent heat release. At the same time, latent heat has an obvious influence on the growth of the crystal interface [11]. To determine the temperature of the growing crystal front, the heat conduction equation can be derived from the enthalpy conservation law in Equation (S10).

$$\frac{\partial T}{\partial t} = \alpha\nabla^2 T + \mathrm{K}\frac{\partial\Psi}{\partial t} \tag{S10}$$

where $\alpha = \kappa_T / (\rho C_P)$; $\mathrm{K} = \Delta H / C_P$; and ρ, C_P, and κ_T represent density, specific heat capacity, and thermal conductivity, respectively.

For the convenience of application, a dimensionless phase-field model is used in the simulation. For this reason, the characteristic length, d, of the parametric single crystal and the mass diffusion coefficient, D, are introduced [12]. Assume that the time and space variables are rearranged to $\hat{t} = Dt / d^2$ and $\hat{\boldsymbol{r}} = \boldsymbol{r} / d$, and the temperature variable is rearranged to $\hat{T} = (T - T_c) / (T_m - T_c)$. From d and D, the interfacial mobility, Γ, is estimated as $\Gamma = D / d^2$ [13]. The dimensionless time step and space step are fixed as $\Delta\hat{t} = 2.5\times10^{-5}$ and $\Delta\hat{x} = \Delta\hat{y} = 1.5\times10^{-2}$, respectively. Thus, the phase-field model can be expressed in the following dimensionless form, as shown in Equations (S11) and (S12):

$$\frac{\partial \Psi(\hat{\boldsymbol{r}},\hat{t})}{\partial \hat{t}} = -\{W\Psi(\Psi-\xi)(\Psi-\xi_0) - \hat{\kappa}_0^2 \hat{\nabla}\cdot(\beta^2(\theta)\hat{\nabla}\Psi) + \hat{\kappa}_0^2 \frac{\partial}{\partial \hat{x}}[\beta(\theta)\beta'_{\Psi_x}(\theta)(\hat{\nabla}\Psi)^2] - \hat{\kappa}_0^2 \frac{\partial}{\partial \hat{y}}[\beta(\theta)\beta'_{\Psi_y}(\theta)(\hat{\nabla}\Psi)^2]\} \tag{S11}$$

$$\frac{\partial \hat{T}}{\partial \hat{t}} = \hat{\alpha}\hat{\nabla}^2 U + \hat{\mathrm{K}}\frac{\partial \Psi}{\partial \hat{t}} \tag{S12}$$

where $\hat{\kappa}_0^2 = \kappa_0^2 / d^2$; $\hat{\alpha} = \alpha / D$; and $\hat{\mathrm{K}} = \mathrm{K}/(T_m - T_c)$ and the barrier of instability energy, ξ, is as follows [10] in Equation (S13).

$$\xi = [\frac{1}{2} + \frac{1}{\pi}\arctan(10\frac{T-T_m}{T_m - T_c})]\hat{\xi} \tag{S13}$$

2. Supplementary Results

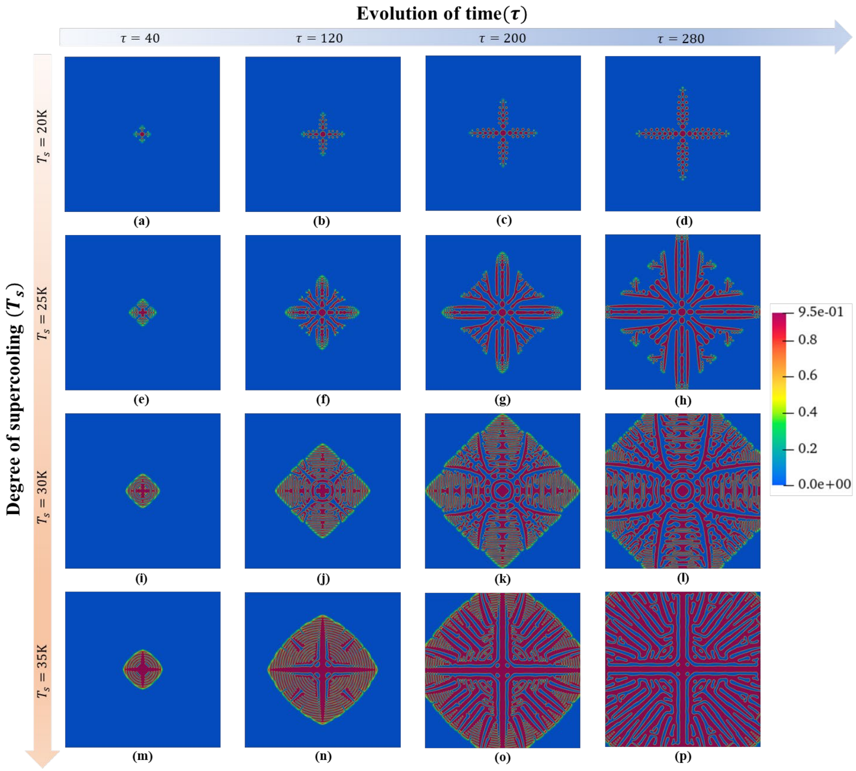

Figure S1. Morphology of PTFE crystal evolution under various supercooling degrees, T_s (anisotropic mode $j = 4$). (**a**–**d**) $T_s = 20$ K, $\tau = 40, 120, 200, 280$; (**e**–**h**) $T_s = 25$ K, $\tau = 40, 120, 200, 280$; (**i**–**l**) $T_s = 30$ K, $\tau = 40, 120, 200, 280$; (**m**–**p**) $T_s = 35$ K, $\tau = 40, 120, 200, 280$.

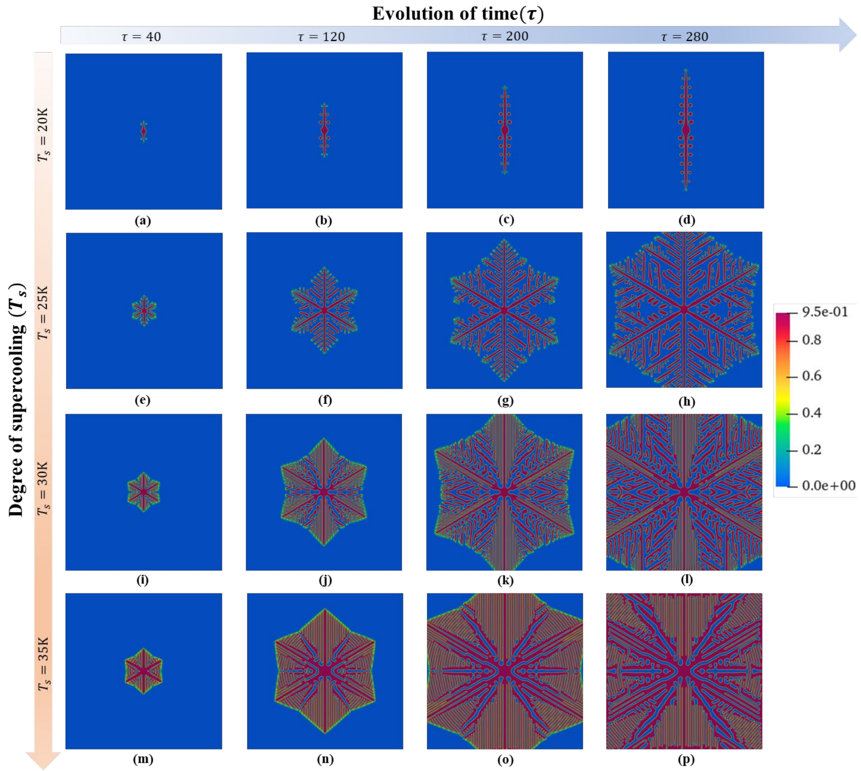

Figure S2. Morphology of PTFE crystal evolution under various supercooling degrees, T_s (anisotropic mode j = 6). (**a–d**) T_s = 20 K, τ = 40, 120, 200, 280; (**e–h**) T_s = 25 K, τ = 40, 120, 200, 280; (**i–l**) T_s = 30 K, τ = 40, 120, 200, 280; (**m–p**) T_s = 35 K, τ = 40, 120, 200, 280.

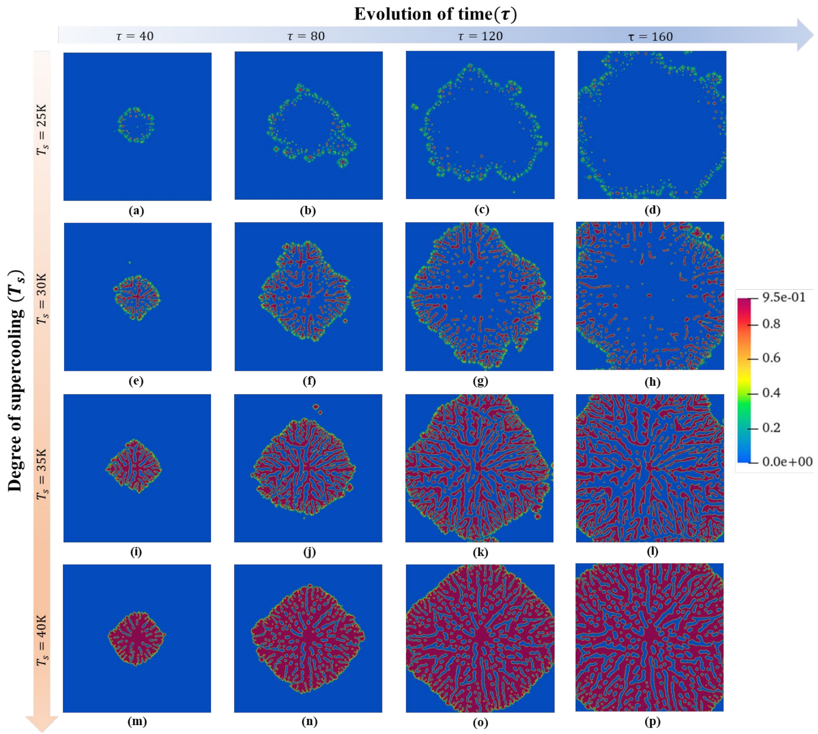

Figure S3. Morphology of PTFE crystal evolution under various supercooling degrees, T_s (anisotropic mode j = 36). (**a–d**) T_s = 25 K, τ = 40, 80, 120, 160; (**e–h**) T_s = 30 K, τ = 40, 80, 120, 160; (**i–l**) T_s = 35 K, τ = 40, 80, 120, 160; (**m–p**) T_s = 40 K, τ = 40, 80, 120, 160.

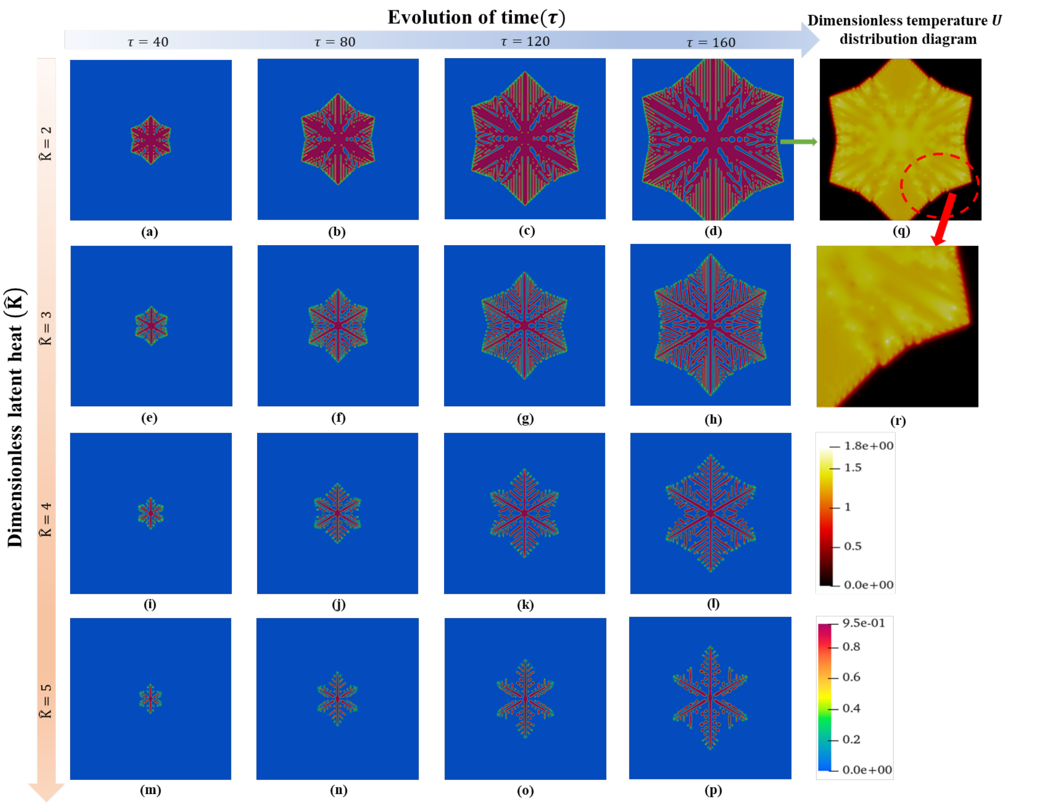

Figure S4. Morphology of PTFE crystal evolution with different dimensionless latent heat, $\hat{K}$. (**a**–**d**) $\hat{K} = 2,\ \tau = 40, 80, 120, 160$; (**e**–**h**) $\hat{K} = 3,\ \tau = 40, 80, 120, 160$; (**i**–**l**) $\hat{K} = 4,\ \tau = 40, 80, 120, 160$; (**m**–**p**) $\hat{K} = 5,\ \tau = 40, 80, 120, 160$; (**q**) dimensionless temperature distribution of Figure S4d; (**r**) local detail of Figure S4q.

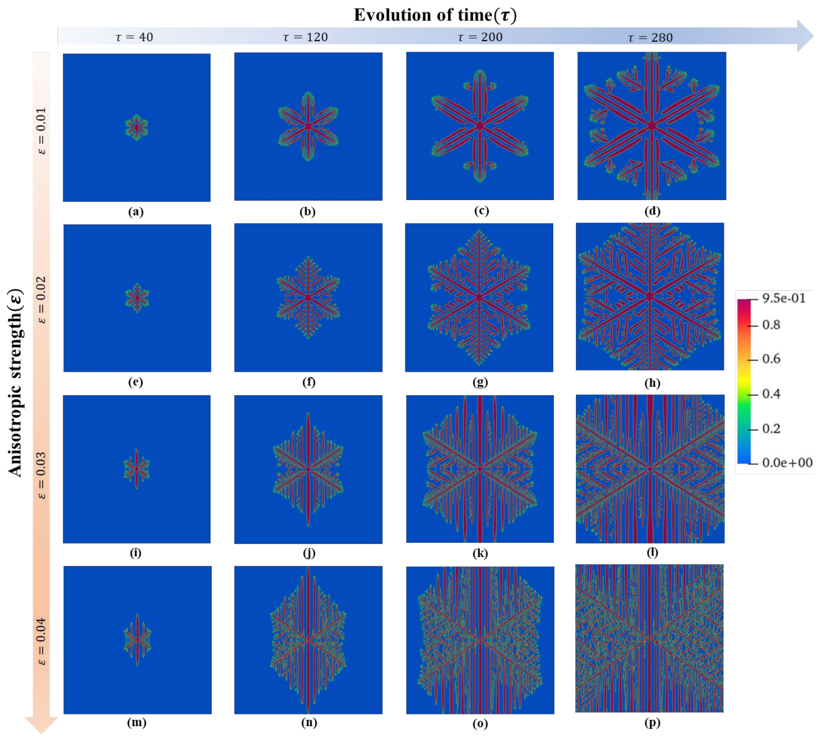

Figure S5. Morphology of PTFE crystal evolution with different anisotropic strengths, ε. (**a**–**d**) ε = 0.01, τ = 40, 120, 200, 280; (**e**–**h**) ε = 0.02, τ = 40, 120, 200, 280; (**i**–**l**) ε = 0.031, τ = 40, 120, 200, 280; (**m**–**p**) ε = 0.04, τ = 40, 120, 200, 280.

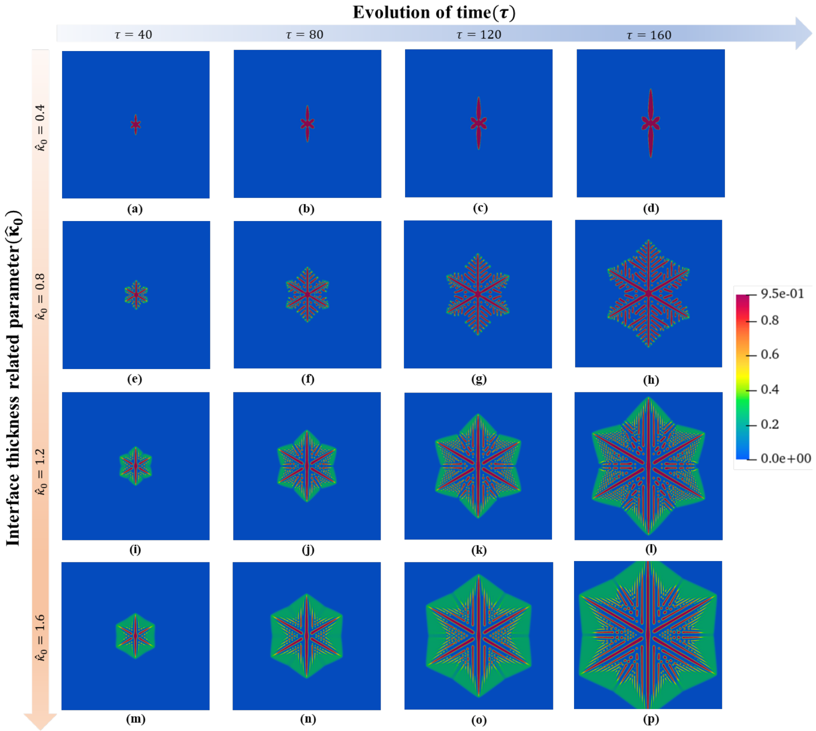

Figure S6. Morphology of PTFE crystal evolution with different interface thickness related parameters, $\hat{\kappa}_0$. **(a–d)** $\hat{\kappa}_0 = 0.4$, $\tau = 40, 80, 120, 160$; **(e–h)** $\hat{\kappa}_0 = 0.8$, $\tau = 40, 80, 120, 160$; **(i–l)** $\hat{\kappa}_0 = 1.2$, $\tau = 40, 80, 120, 160$; **(m–p)** $\hat{\kappa}_0 = 1.6$, $\tau = 40, 80, 120, 160$.

Supplementary References

[1] Hohenberg, P.C.; Halperin, B.I. Theory of dynamic critical phenomena. Rev. Mod. Phys. **1977**, 49, 435.

[2] Landau, L.D. The theory of phase transitions. Nature **1936**, 138, 840-841.

[3] Xu, H.; Matkar, R.; Kyu, T. Phase-field modeling on morphological landscape of isotactic polystyrene single crystals. Phys. Rev. E **2005**, 72, 011804.

[4] Hohenberg, P.C.; Krekhov, A.P. An introduction to the Ginzburg-Landau theory of phase transitions and nonequilibrium patterns. Phys. Rep.-Rev. Sec. Phys. Lett. **2015**, 572, 1-42.

[5] Costa, A.R.D.M.; Santos, R.M.; Ito, E.N. Melt and cold crystallization in a poly (3-hydroxybutyrate) poly (butylene adipate-co-terephthalate) blend. J. Therm. Anal. Calorim. **2019**, 137, 1341-1346.

[6] Harrowell, P.R.; Oxtoby, D.W. On the interaction between order and a moving interface: Dynamical disordering and anisotropic growth rates. J. Chem. Phys. **1987**, 86, 2932-2942.

[7] Xu, H.; Matkar, R.; Kyu, T. Phase-field modeling on morphological landscape of isotactic polystyrene single crystals. Phys. Rev. E **2005**, 72, 011804.

[8] Hoffman, J.D.; Weeks, J.J. Melting process and the equilibrium melting temperature of polychlorotrifluoroethylene. J. Res. Natl. Bur. Stand., Sect. A **1962**, 66, 13-28.

[9] Housmans, J.W.; Peters, G.; Meijer, H. Flow-induced crystallization of propylene/ethylene random copolymers. J. Res. Natl. Inst. Stand. Technol. **2009**, 98, 693-705.

[10] Wang, X.D.; Ou, Y.J.; Su, J. A phase-field model for simulating various spherulite morphologies of semi-crystalline polymers. Chin. Phys. B **2013**, 22, 106103.

[11] Schultz, J.M. Self-generated fields and polymer crystallization. Macromolecules **2012**, 45, 6299-6323.

[12] Xu, H.; Matkar, R.; Kyu, T. Phase-field modeling on morphological landscape of isotactic polystyrene single crystals. Phys. Rev. E **2005**, 72, 011804.

[13] Wang, X.D.; Zhang, H.X.; Zhou, W. A 3D phase-field model for simulating the crystal growth of semi-crystalline polymers. Int. J. Heat Mass Transf. **2017**, 115, 194-205.
